# A Lightweight Network for Free Fluid Detection in Focused Assessment with Sonography in Trauma (FAST) Examination

**DOI:** 10.3390/bioengineering13050550

**Published:** 2026-05-13

**Authors:** Mingyi Yang, Nianzeng Yuan, Shipeng Han, Tianjiao Guo, Wen Luo, Hao Lv

**Affiliations:** 1 School of Biomedical Engineering, Air Force Medical University, Xi’an 710032, China; mingyi_yang@fmmu.edu.cn (M.Y.);; 2 Department of Ultrasound, Xijing Hospital of Air Force Medical University, Xi’an 710032, China

**Keywords:** free fluid detection, lightweight network, Focused Assessment with Sonography in Trauma (FAST)

## Abstract

The Focused Assessment with Sonography in Trauma (FAST) enables rapid point-of-care screening for internal hemorrhage by detecting free fluid, but its accuracy is highly operator-dependent and prone to missed diagnoses in emergency settings. While deep learning–based AI assistance can address these limitations, most existing models rely on computationally intensive networks, restricting deployment on resource-limited bedside devices. Thus, developing a lightweight architecture for free fluid detection is essential for clinical translation. We propose a lightweight model based on YOLOX, incorporating a dual-stream fusion (DSF) backbone to preserve spatial details while reducing computation, and a global fusion feedback (GFF) neck to enhance efficient multi-scale feature fusion. We also built a dedicated dataset using ultrasound images from rabbits with active liver hemorrhage to better mimic in vivo sonographic features of free fluid. Compared with mainstream detectors, our method achieves the lowest FLOPs and parameter count while maintaining superior precision, recall, and F1-score. Ablation studies validate that DSF improves accuracy and reduces complexity, and GFF further lowers computational costs with minimal performance tradeoff. The proposed approach enables fast, accurate free fluid detection on constrained bedside devices, advancing intelligent point-of-care trauma assessment.

## 1. Introduction

In acute trauma care, internal hemorrhage is one of the leading causes of mortality [1]. More than 60% of blunt internal trauma cases occur in a pre-hospital setting, such as traffic accidents and combat injuries [2]. Consequently, the accuracy of rapid point-of-care assessment directly influences clinical decision-making during the critical “golden hour” of resuscitation [3]. Computed Tomography (CT) is traditionally regarded as the gold standard for diagnosing abdominothoracic injuries [4]. However, the inherent limitations of CT imaging, including site requirements and radiation exposure, often impede timely evaluation in patients with suspected internal hemorrhage [5]. Within contemporary trauma resuscitation protocols, point-of-care ultrasound has become the first-line imaging modality for rapid detection of hemoperitoneum in emergency and critical care settings [6]. The Focused Assessment with Sonography for Trauma (FAST) [7] examination systematically detects free fluid in the pericardial sac and peritoneal cavity. When combined with clinical judgment, this bedside diagnostic technique helps refine diagnoses in trauma patients and guides decisions regarding the need for emergent surgical intervention or transfer to trauma centers. Moreover, ultrasound imaging enables clinicians to rapidly identify life-threatening injuries at the bedside or in resource-limited environments without exposing patients to radiation [8].

Although FAST examination has been widely adopted in some hospitals, its further development remains constrained by significant limitations. A major challenge lies in the fact that ultrasound imaging is highly operator-dependent, requiring substantial training and clinical experience [9]. Yet, across various clinical settings, including emergency scenes and resource-limited areas, there is a widespread shortage of adequately trained and experienced medics [10]. In acute trauma care, variability in operator skill levels can lead to missed life-threatening injuries. Even medics who have received point-of-care ultrasound training may not always be able to accurately interpret ultrasound images promptly in every situation. This issue is exacerbated in emergencies, where the need for rapid assessment increases the pressure on the operator.

Given these challenges, deep learning (DL) technology demonstrates considerable potential. During FAST examination conducted by paramedics under urgent conditions, AI-assisted ultrasonography can improve diagnostic performance by enabling rapid identification and localization of free fluid, thereby shortening examination time and accelerating clinical intervention. Furthermore, this technology could potentially allow medics without specialized diagnostic training to make effective clinical decisions using ultrasound devices. Currently, deep learning models have been widely applied in FAST examination [11,12,13,14,15,16,17,18]. However, despite achieving promising accuracy, most of these models adopt generic object detection architectures and incur considerable computational costs. However, point-of-care and pre-hospital ultrasound devices are severely resource-constrained, which poses a major obstacle to the clinical application of intelligent diagnosis. Consequently, existing AI-assisted free fluid detection methods have remained largely in the research domain, with no practical deployment pathway for point-of-care use. Furthermore, the datasets used in these studies primarily consist of effusions (e.g., pleural or ascitic fluid), which do not necessarily represent the dynamic, irregular morphology of free fluid resulting from active internal hemorrhage.

To bridge this gap, we propose a lightweight detection network specifically designed for free fluid identification in FAST examinations, targeting resource-constrained bedside devices. The network is built upon the efficient YOLOx [19] architecture, with two key structural innovations: a Dual-Stream Fusion (DSF) backbone that decomposes features into high- and low-frequency components to preserve spatial details while reducing computation, and a Global Fusion Feedback (GFF) neck that integrates multi-scale features with bottom-up refinement to improve fusion efficiency.

Another distinctive aspect of our work is the training dataset. Unlike prior studies that used static effusion data, we collected and annotated ultrasound images from rabbits with actively hemorrhaging liver tissue. This allows our model to learn the morphological characteristics of free fluid during active hemorrhage, which is a more clinically relevant and challenging scenario.

Experiments show that our lightweight design achieves competitive detection accuracy while reducing computational complexity by an order of magnitude compared with mainstream detectors. This makes accurate, automated internal hemorrhage assessment feasible on portable ultrasound devices without requiring cloud connectivity or high-end hardware—thereby providing a practical technical solution for point-of-care intelligent internal hemorrhage detection. The code is available at https://github.com/Mingyi-Yang-hub/lightweight-detection (accessed on 9 May 2026).

The remainder of this paper is organized as follows. Section 2 reviews the related work, covering free fluid detection and lightweight object detection methods. Section 3 elaborates on the proposed method in detail, including network architecture design, dataset construction, and model training strategy. Section 4 presents the experimental results and analysis, involving comparative evaluation with mainstream methods as well as ablation experiments. Section 5 provides relevant discussions on this work. Finally, the overall conclusions are drawn in Section 6.

## 2. Related Work

### 2.1. Free Fluid Detection

In 2016, a study from Boston University conducted a pilot study to explore the feasibility of automatically detecting abdominal free fluid during FAST examination [11]. Features related to geometric properties, grayscale color attributes, and edge sharpness were extracted from the images. These features were then normalized and used as inputs to a support vector machine classifier to determine whether each hypoechoic region of interest was free fluid (positive) or non-free fluid (negative). However, the features extracted by traditional methods had a limited capacity to represent free fluid, thus restricting classification accuracy.

With advances in deep learning, Cheng et al. [12] pioneered the application of a ResNet50-V2 [20] network to detect the free fluid in ultrasound images. This end-to-end approach leveraged the network’s capability to autonomously extract hierarchical features from the ultrasound images for classifying scans based on the presence or absence of free fluid. Separately, research from the University of California [13] focuses on classifying FAST exam views in pediatric patients, while another model [14] is dedicated to identifying pleural effusion in lung ultrasound across different clinical settings. Nonetheless, these studies primarily address the classification of free fluid presence without localizing it precisely. In contrast, Leo et al. [15] developed a deep learning algorithm to identify both the presence and location of free fluid using POCUS, assisting novice clinicians in interpreting FAST exams accurately. Their algorithm is based on YOLOv3 [21], a model well-regarded for its object detection performance across multiple domains. Similarly, Lkay et al. utilized YOLOv3 to detect pericardial effusion—free fluid in the cardiac region [16]. To deliver more precise delineation of free fluid areas, Lin et al. [17] and Huang et al. [18] adopted U-Net [22] and Attention U-Net [23], respectively, for segmenting free fluid in ultrasound images.

In existing research, methods for detecting free fluid in ultrasound images primarily focus on verifying the basic feasibility of deep learning techniques for this task. Consequently, most approaches directly employ general-purpose deep learning models for implementation. While these models demonstrate strong generalization capabilities across various visual tasks, their structures are typically large with a large number of parameters and high computational overhead. However, the practical application scenarios for free fluid detection are often concentrated in point-of-care ultrasound examinations. Under such conditions, directly deploying complex general-purpose models may lead to slow inference speeds, inadequate device responsiveness, and difficulty in meeting the requirements for real-time clinical detection.

### 2.2. Lightweight Methods

In the medical field, limited by computing power, power consumption, and storage capacity, lightweight deep learning networks have become a research focus. For instance, a lightweight model named EpiBrCan-Lite is designed for the classification of breast cancer subtypes using DNA methylation data [24]. Chen et al. [25] introduce an adapter-enhanced DINOv3 for automated ischemic stroke lesion segmentation. Portable ultrasound diagnostic equipment has stricter hardware constraints, imposing higher requirements on the model size and computational complexity of deep learning algorithms. Corresponding research has been carried out. For instance, Pang et al. [26] propose a lightweight approach for left ventricle segmentation in echocardiography. Lu et al. [27] developed a lightweight multi-task framework for medical image segmentation and landmark localization.

In lightweight networks, the YOLO series is widely applied in medical ultrasound detection due to its efficiency. YOLOv1 [28] enabled end-to-end real-time detection, laying a foundation for clinical deployment. YOLOv3 [21] introduced FPN to enhance multi-scale feature fusion and small lesion detection and has been applied in thyroid and breast ultrasound. YOLOv4 [28] and YOLOv5 [29] further improved performance, with YOLOv5 offering scalable models to fit portable ultrasound devices. YOLOv8 [30] adopted the C2f module but still has limited lightweight potential due to complex structures. The latest version, YOLO26 [31], used an NMS-free end-to-end architecture and achieved higher accuracy. As a milestone, YOLOx [19] strikes a better balance between performance and lightweight design. With an anchor-free and decoupled head, it has a simple, modular structure that maintains accuracy while allowing flexible, lightweight optimization. Its nano variant, in particular, has extremely low parameters and computation, making it ideal for resource-constrained clinical settings.

Although the above lightweight YOLO models perform well in medical ultrasound detection, none are specially designed for free fluid detection. Ultrasound-free fluid exhibits unique features, typically appearing as irregular anechoic or hypoechoic regions with position and morphology varying with patient posture, and it is often small, scattered, and easily disturbed by ultrasound artifacts and complex tissues. Therefore, a dedicated lightweight detection network tailored to the characteristics of free fluid is required to enable real-time and accurate clinical detection.

## 3. Methods

This section provides a systematic exposition of the proposed method. First, Section 3.1 details the model structure, explaining its core components and design principles. Subsequently, Section 3.2 describes the acquisition process of the dataset employed in this study. Then, Section 3.3 outlines the specific implementation strategy for training the network. Finally, Section 3.4 introduces the quantitative metrics used to evaluate the model’s performance.

### 3.1. Model Structure

**Figure 1:**
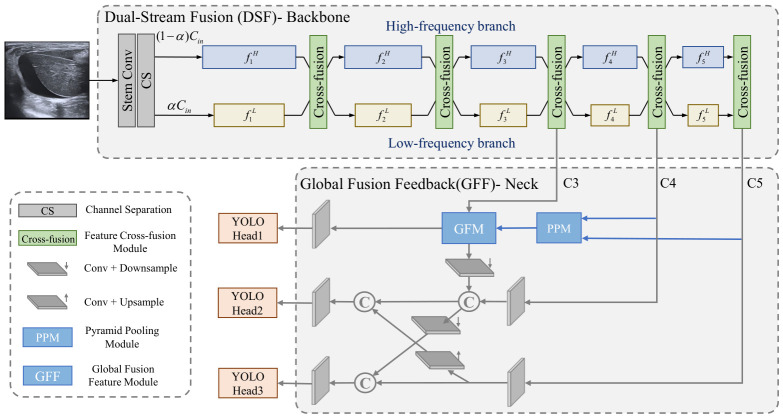
The structure of the proposed lightweight network for free fluid detection.

The overall structure of the proposed network is illustrated in Figure 1. Built upon the YOLOx baseline, an efficient framework for object detection, we introduce targeted optimizations in the backbone and neck components to better adapt to the characteristics of free fluid in ultrasound images. These modifications aim to reduce network complexity while preserving detection accuracy, thereby enhancing overall performance in this medical imaging context. To fully extract features of free fluid regions with lower computational cost, the backbone module (detailed in Section 3.1.1) decomposes features into two parallel pathways, i.e., high-frequency and low-frequency. The low-frequency branch operates at a smaller resolution to reduce computational load, while the high-frequency branch supplements detailed information, thereby balancing efficiency and representational capacity. Simultaneously, a feature cross-fusion module is introduced to facilitate complementary interaction between the two branches, which enhances feature expressiveness and mitigates information loss. Furthermore, to enhance multi-scale feature fusion while maintaining low complexity, the neck module (detailed in Section 3.1.2) introduces a structure based on a global fusion feature. This architecture enables top-down propagation of high-level semantic information through the global fusion feature while reducing redundancy along the feature transmission path. Simultaneously, the fused global information is fed back to each feature level, strengthening bottom-up detail representation and improving the robustness of multi-scale features.

#### 3.1.1. Dual-Stream Fusion Backbone (DSF-Backbone)

**Figure 2:**
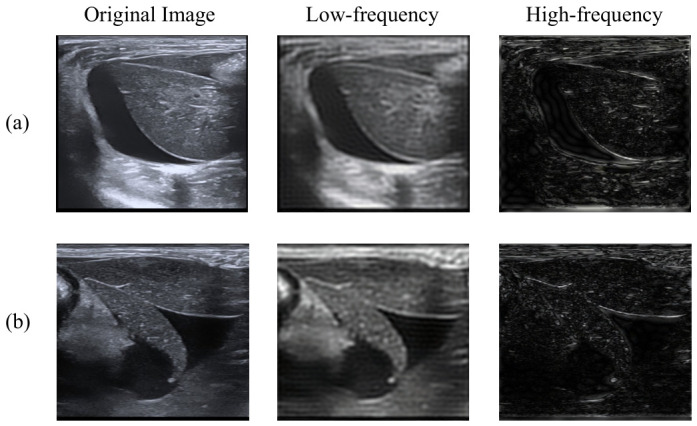
Visualization of frequency decomposition of ultrasound image with free fluid. (**a**,**b**) illustrate two sample cases.

In ultrasound imaging, free fluid typically appears as hypoechoic areas with continuous boundaries due to the low attenuation of sound waves of fluids. This characteristic exhibits a distinct pattern in the spatial frequency domain: the overall grayscale distribution and regional continuity are primarily contained in the low-frequency components of the image, while boundary details and textural features are carried by the high-frequency components. To more clearly observe the information characteristics across different frequency bands, we performed visualization by conducting frequency-domain decomposition analysis on ultrasound images containing free fluid. The corresponding low-frequency and high-frequency components are shown in Figure 2. Systematic observation reveals that the low-frequency components significantly retain the overall echo-intensity distribution characteristics of the region, clearly displaying the macroscopic morphology and average grayscale level of the free fluid area, which aligns with the physical imaging mechanism of the hypoechoic nature of fluid. As shown in Figure 2, the location and extent of the hypoechoic area can be largely identified using only the low-frequency reconstructed image. The high-frequency components primarily represent detailed image information, including key diagnostic features such as boundary sharpness and internal texture. Boundary sharpness aids in accurately defining the extent of free fluid areas, while the textural features displayed in the high-frequency components effectively distinguish parenchymal tissue areas. Another important basis for identifying free fluid is that it is often surrounded by parenchymal tissue structures. Therefore, high-frequency information holds significant value in assisting localization and differential diagnosis.

Based on the above analysis, we designed a dual-frequency branch multi-scale feature extraction backbone network, whose structure is illustrated in the “Backbone” part of Figure 1. The network first employs the stem convolutions (Stem Conv) in YOLOx to perform preliminary feature extraction on the original ultrasound images. The resulting features are then split into two independent branches along the channel dimension in proportions $\alpha_{in}$ and $\left(1-\alpha_{in}\right)$. One branch focuses on low-frequency feature extraction, capturing the macroscopic morphology and overall grayscale distribution in the image. Due to the lower resolution of feature maps in the low-frequency branch, it significantly reduces the model’s computational complexity and memory usage, thereby improving the network’s computational efficiency. The other branch specializes in high-frequency feature extraction, capturing fine structural details such as edges and internal textures, which serve as supplementary information, enabling the network to obtain more comprehensive and accurate image features.

**Figure 3:**
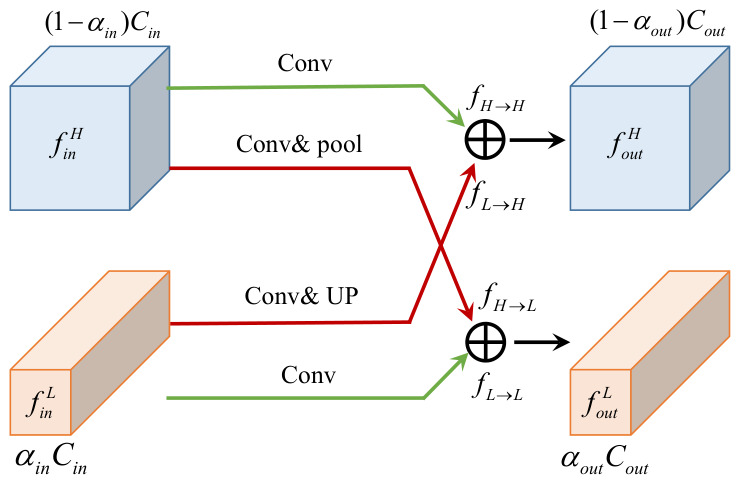
The structure of feature cross-fusion module.

For a single branch, as the feature extraction module deepens, features on that branch gradually lose some information and become overly simplified. To address this issue, we introduce a feature cross-fusion module after each scale of feature extraction, enabling information exchange and fusion between the high-frequency and low-frequency features of the two branches. Inspired by octave convolution [32], the structure of the cross-fusion module is designed based on octave convolution, with the specific architecture illustrated in Figure 3. The dual-frequency branches process the high-frequency features ($f_{in}^{H}$) and low-frequency features ($f_{in}^{L}$), respectively. After interaction and fusion, the generated new high-frequency features ($f_{out}^{H}$) and low-frequency features ($f_{out}^{L}$) are given by

(1) $$f_{out}^{H}=f_{L\to H}+f_{H\to H},$$

(2) $$f_{out}^{L}=f_{H\to L}+f_{L\to L},$$

respectively, where $f_{A\to B}$ denotes the feature maps obtained by the convolutional update from feature map group *A* to group *B*. Specifically, $f_{H\to H}$ and $f_{L\to L}$ represent the feature maps obtained through intra-frequency updates, while $f_{L\to H}$ and $f_{H\to L}$ represent the feature maps obtained through inter-frequency communication. Among them, the intra-frequency update is implemented via a convolution operation; the inter-frequency communication corresponding to $f_{H\to L}$ is completed through convolution and pooling-based downsampling operations (Conv & pool), and $f_{L\to H}$ is realized through convolution and upsampling operations (Conv & Up). By leveraging octave convolution, cross-frequency communication is effectively achieved, promoting complementary information integration between the two branches.

#### 3.1.2. Global Fusion Feedback Neck (GFF-Neck)

In object detection networks, the neck module plays a crucial role in feature fusion, with its core objective being the comprehensive integration of multi-scale features extracted from the backbone module. Typically, the outputs from the last three hierarchical levels of the backbone module are selected for fusion, such as the lower-level C3, mid-level C4, and highest-level C5, as shown in Figure 1. These feature maps possess varying spatial resolutions, containing rich positional details, mid-level semantic information, and higher-level abstract features, respectively, collectively forming the foundation of multi-scale representation for detection tasks. Currently, widely used neck modules, such as the Feature Pyramid Network (FPN) [33] and its extensions like the Path Aggregation Network (PANet) [34], employ bidirectional top-down and bottom-up fusion mechanisms across layers to achieve thorough interaction among cross-scale features. This effectively enhances the model’s ability to recognize objects at multiple scales. However, such methods often introduce significant computational and parameter overhead, and feature propagation paths may contain certain redundancies, which limit the further balance between efficiency and performance. Therefore, with the aim of improving computational efficiency while preserving effective feature fusion, we explore a more efficient feature fusion mechanism, attempting to achieve effective integration of multi-scale features while reducing redundant operations.

**Figure 4:**
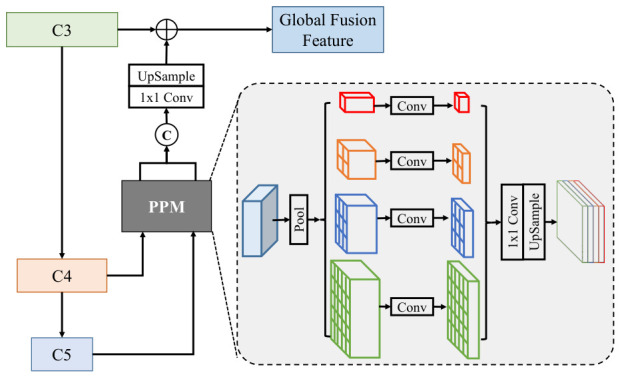
The structure of Global Fusion Module (GFM) and Pyramid Pooling Module (PPM).

To reduce redundancy in feature propagation paths, we first propose a global fusion mechanism that directly integrates multi-scale features into a unified global fusion (GF) feature. This GF feature is subsequently leveraged to enhance the expressive capacity of all feature levels, thereby streamlining propagation paths and reducing redundancy. The generation of the GF feature consists of two main steps: first, compressing high-level semantic features (C4/C5) to extract lightweight contextual descriptors; then, fusing them with the detail-rich low-level feature C3, as illustrated in Figure 4. Specifically, for the C4/C5 features, a Pyramid Pooling Module (PPM) [35] is applied to generate multi-receptive-field contextual feature maps through adaptive average pooling at four different scales: 1×1, 2×2, 3×3, and 6×6. The outputs from the PPM undergo 1×1 convolution and upsampling operations, followed by concatenation and fusion along the channel dimension. The resulting features are then processed through convolution and upsampling and finally combined with the C3 feature to form the GF feature. This approach offers several advantages:

➀ The pooling operations at different scales in the PPM capture a range of contextual information. By integrating multi-scale features, the model’s receptive field coverage is effectively expanded, which aids in improving the network’s ability to identify target regions.
➁ The pooling operations themselves are parameter-free, significantly reducing computational overhead.
➂ This design avoids the traditional stepwise propagation structure in FPN, such as C5 → C4 → C3, reducing the number of feature transmissions and mitigating information degradation during propagation.

The GF feature is then further fed back to each feature level from the bottom up and integrated with their original features, as illustrated in the “Neck” part of Figure 1. The fused features are then input into their corresponding detection heads to accomplish the final detection tasks. This design effectively combines global contextual information with local detail features while maintaining a bidirectional information flow (top-down and bottom-up): on one hand, the global information fusion mechanism enables the top-down propagation of high-level semantic information; on the other hand, by feeding GF features back to each level, it further enhances the bottom-up expression of detailed information. Although this process is simple, it enriches and reinforces the feature representation at each level, achieving efficient multi-scale feature fusion.

### 3.2. Data Collection

Taking into account the practical challenges in obtaining sufficient data on active internal hemorrhage in humans in order to accurately simulate the ultrasound characteristics of free fluid in the state of internal hemorrhage, this study established an active liver bleeding model in rabbits. This animal model is used to collect ultrasound images under controlled and standardized conditions, creating a dedicated dataset to validate the effectiveness of the proposed detection method. The dataset, derived from real active hemorrhage processes, better reflects the ultrasonographic features of traumatic internal hemorrhage. Additionally, this dataset can be used to verify the model’s efficacy and lay the foundation for future translation of the algorithm to human applications.

**Figure 5:**
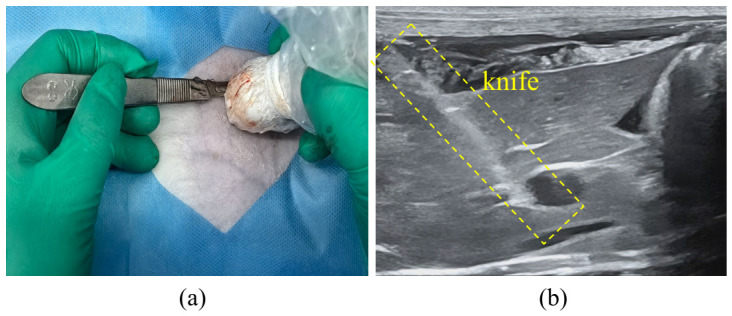
Establishment of the active hemorrhage model under ultrasound guidance. (**a**) Intraoperative photograph during model preparation; (**b**) Corresponding ultrasound image acquired during modeling.

In the experiments, 18 healthy adult rabbits were selected. Under ultrasound guidance, the anatomical position of the liver was precisely located, and an active hemorrhage model was constructed by puncturing the liver parenchyma with an 11-gauge sharp knife while avoiding major blood vessels and bile ducts, as shown in Figure 5. Ultrasound image acquisition commenced immediately after inducing hemorrhage, and the ultrasound images of the liver and the kidney-surrounding area were recorded from multiple perspectives. A total of 677 frames were extracted at fixed intervals from the recorded ultrasound videos to form the free fluid dataset. During the acquisition process, free fluid was absent in some images due to probe movement or positioning deviations. Such images were retained during dataset construction and annotated as target-free samples to enhance the model’s ability to identify negative samples and improve the overall robustness of the network. All data were professionally annotated by physicians with over five years of clinical experience to obtain accurate labels for the free fluid.

### 3.3. Model Training

All models in this study were implemented using the PyTorch framework and trained on an NVIDIA RTX 3090 GPU. The model was optimized using Stochastic Gradient Descent (SGD) with a momentum of 0.9 and a weight decay coefficient of $5\times 10^{-4}$ to improve generalization. The training process employed a multi-stage learning rate scheduling strategy. Specifically, the initial value of the learning rate was set to $1\times 10^{-2}$ and gradually increased to this value over the first 5 epochs using a quadratic formula warm-up strategy. Subsequently, the learning rate was reduced to $1\times 10^{-3}$ and $1\times 10^{-4}$ at the 60% and 85% marks of the total training epochs, respectively, to facilitate convergence toward a better local optimum in the later training stages. Training was performed with a batch size of 16. All input images were resized to 640×640 pixels, and data augmentation techniques were applied to enhance model robustness.

The loss function is designed following YOLOx’s unified framework, which integrates object detection and classification into a single objective. The overall loss can be expressed as

(3) $$L_{\mathrm{total}}=\lambda_{\mathrm{reg}}L_{\mathrm{reg}}+\lambda_{\mathrm{cls}}L_{\mathrm{cls}}+\lambda_{\mathrm{conf}}L_{\mathrm{conf}},$$

where $\lambda_{\mathrm{reg}}$, $\lambda_{\mathrm{cls}}$, and $\lambda_{\mathrm{conf}}$ are balancing coefficients, which are assigned values of 5, 1, and 1, respectively, in our experiments according to the default settings of YOLOx. Each loss component is defined as follows: (1) Bounding box regression loss ($L_{\mathrm{reg}}$), which uses IoU Loss to improve localization precision; (2) Class prediction loss ($L_{\mathrm{cls}}$) and object confidence loss ($L_{\mathrm{conf}}$), both implemented using Cross Entropy, with a weighted positive–negative sample strategy to alleviate class imbalance; (3) Instead of static assignment, SimOTA dynamically selects positive samples based on the matching cost between predictions and ground truths. The matching cost for the *i*-th prediction and *j*-th ground truth is defined as:

(4) $$COST_{ij}=\omega_{1}\cdot L_{\mathrm{cls}}^{\left(ij\right)}+\omega_{2}\cdot L_{\mathrm{reg}}^{\left(ij\right)},$$

where $\omega_{1}$ and $\omega_{2}$ are adjustable weights, which are assigned values of 1 and 3, respectively, in our experiments according to the default settings of YOLOx. Based on $COST_{ij}$, the top-*k* predictions with the lowest cost are assigned as positive samples for each ground truth, which improves both training efficiency and final detection performance.

Given the limited scale of the dataset, data augmentation was employed during training to enhance the model’s generalization ability. We adopt the data augmentation methods used in YOLOx, including Mosaic, MixUp, and RandomFlip. It should be noted that RandomFlip is applied only horizontally, not vertically. This is because ultrasound images exhibit a distinct physical orientation during acquisition: the upper part of the image corresponds to the epidermal tissue near the probe, while the lower part typically represents acoustic shadows or artifact regions. Vertical flipping would disrupt the alignment between the image structure and the actual anatomical position, violating the physical principles of ultrasound imaging. Therefore, vertical flipping is not adopted in this study.

### 3.4. Evaluation Metrics

To comprehensively evaluate the model and compare it with other mainstream object detection models, we employ precision, recall, mean average precision (mAP), and F1-score as performance metrics. Higher recall indicates a lower risk of missed detection, while higher precision corresponds to a lower false alarm rate; the F1-score provides a balanced measure of both. In addition, model complexity is assessed using the number of parameters (Params) and gigaflops operations per second (FLOPs).

Given that the free fluid detection task places relatively relaxed demands on bounding box localization accuracy while prioritizing the avoidance of missed and false detections, this study reports not only the commonly used mAP@50:95 in object detection (which reflects overall performance across IoU thresholds from 0.5 to 0.95 with a step size of 0.05) but also fine-grained metrics under different IoU strictness levels. Specifically, precision, recall, and F1-score are calculated separately at IoU thresholds of 0.5 (relatively loose matching) and 0.75 (relatively strict matching).

Specifically, the following metrics are reported:

mAP@50:95: The mean average precision computed over IoU thresholds from 0.5 to 0.95 with a step size of 0.05, reflecting the model’s overall robustness in localization quality across varying matching strictness.

Metrics at IoU = 0.5: precision, recall, and F1-score under a looser matching condition.

Metrics at IoU = 0.75: precision, recall, and F1-score under a stricter matching condition.

Due to the limited sample size of the dataset, a 5-fold cross-validation strategy was adopted to fully evaluate model performance and enhance result stability. All data were randomly divided into five mutually exclusive subsets. In each validation round, one subset was used as the test set while the remaining four formed the training set, resulting in five independent training-test cycles. To ensure fairness and consistency across comparative experiments, the same set of data partitions was used in all comparisons, thereby eliminating performance variations caused by random data splits. All reported performance metrics are the arithmetic mean of the results from the five folds. This approach provides a more robust estimate of the model’s generalization ability under data-limited conditions and reduces evaluation bias introduced by single random partitioning.

## 4. Results

### 4.1. Comparison with Mainstream Object Detectors

Existing methods for free fluid detection commonly employ mainstream detection models. To validate the effectiveness of the proposed method against these mainstream models, we select several mainstream networks for comparative experiments, including the two-stage detector Faster R-CNN [36] and the single-stage detectors SSD [37] and FCOS [38]. In addition, representative models from the YOLO series are adopted in this paper for comparative analysis and reference, including YOLOv3, YOLOx, and YOLO26-n (the most lightweight version of YOLO26 [31]). YOLOx is available in several versions with different scales, which exhibit distinct differences in model complexity. Therefore, YOLOx-s, YOLOx-tiny, and YOLOx-nano are selected for experiments, with their model complexity decreasing sequentially. Furthermore, the proposed lightweight improvement design is implemented based on YOLOx-tiny and YOLOx-nano, and the improved models are denoted as YOLOx-tiny-Lite and YOLOx-nano-Lite, respectively. All compared models adopt the same training strategy, data augmentation scheme, and dataset to ensure consistent experimental conditions and enable a fair evaluation of each model’s performance.

**Figure 6:**
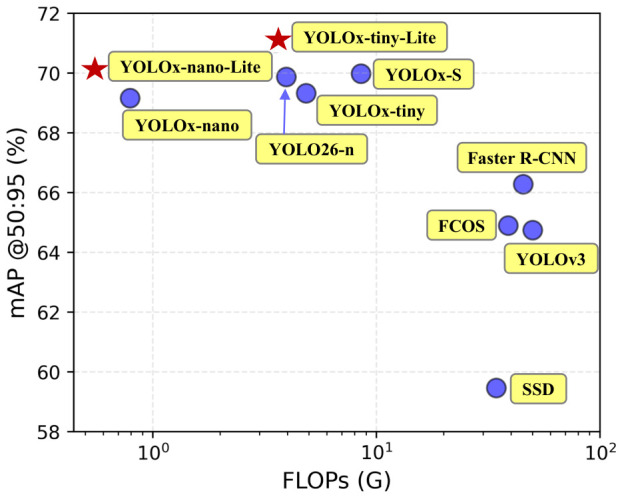
Comparison of FLOPs and mAP@50:95 across different methods.

Figure 6 presents a comparison of FLOPs and mAP@50:95 among different models. It can be seen that the YOLO series models generally have lower computational cost and higher detection accuracy, yielding better overall performance than other comparative methods. Within the YOLO series, the proposed lightweight improvement method achieves performance gains over its corresponding baseline models. Specifically, YOLOx-tiny-Lite versus YOLOx-tiny and YOLOx-nano-Lite versus YOLOx-nano both reduce FLOPs while improving the mAP@50:95 metric, which verifies the effectiveness of the proposed lightweight scheme. Furthermore, among all compared models, only YOLOx-tiny-Lite and YOLOx-nano-Lite achieve an mAP@50:95 higher than 70%, with both maintaining low model complexity. The specific values are presented in Table 1.

Table 1 provides more detailed performance comparisons of different models, including precision, recall, and F1-score under various IoU thresholds, as well as FLOPs and Params. At an IoU threshold of 0.5, all tested methods achieve high precision and recall, both exceeding 95%. This indicates that all models can effectively detect free fluid with high accuracy when the requirements for bounding box localization are relatively relaxed. Among them, YOLOx-tiny-Lite obtains the highest F1-score, demonstrating the optimal balance between precision and recall. YOLOx-nano-Lite also achieves a higher F1-score than YOLOx-nano. At an IoU threshold of 0.75, YOLOx-tiny-Lite and YOLOx-nano-Lite exhibit more advantageous performance than other compared models, with higher precision, recall, and F1-score than other methods, while maintaining low model complexity. This shows that they can identify free fluid and provide its corresponding position more accurately when stricter requirements are imposed on detection box localization accuracy.

**Figure 7:**
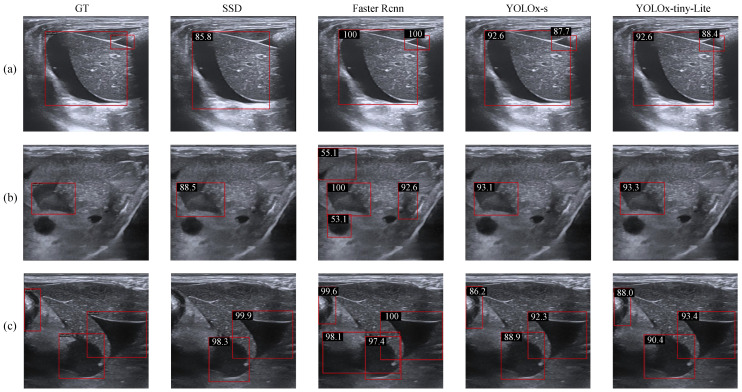
Visual comparison of the detection results from different methods. (**a**–**c**) illustrate three sample cases. Red boxes indicate the detection results.

Figure 7 presents a visual comparison of detection results among SSD, Faster R-CNN, YOLOx-s, and YOLOx-tiny-Lite. As illustrated in the three representative cases—(a), (b), and (c)—SSD exhibits instances of missed detection. Faster R-CNN, while demonstrating high prediction confidence in clearly distinguishable regions, is susceptible to misclassification. For example, in (b), the hypoechoic circular region in the lower-left corner corresponds anatomically to a blood vessel; however, Faster R-CNN incorrectly identifies it as free fluid with a confidence score of 53.1%. The detection accuracy of YOLOx-tiny-Lite is comparable to that of YOLOx-s, while its lower computational complexity constitutes an advantage.

### 4.2. Ablation Studies

#### 4.2.1. Optimal Channel Allocation Ratio in DSF-Backbone

In the Dual-Branch Fusion Backbone, the feature channels are partitioned into two pathways: high-frequency and low-frequency. The proportion of channels allocated to the low-frequency branch is denoted as $\alpha_{in}$, while the high-frequency branch occupies the remaining $\left(1-\alpha_{in}\right)$ of the channels. To investigate the effect of the channel allocation ratio on model performance, we took YOLOx-tiny-Lite as the benchmark and systematically varied $\alpha_{in}$ and conducted a series of comparative experiments. Generally, $\alpha_{in}$ and $\alpha_{out}$ are equal. For brevity, we adopt a unified parameter α to represent both $\alpha_{in}$ and $\alpha_{out}$ for conciseness. The results are presented in Table 2.

The experimental results demonstrate that as the proportion of low-frequency channels α increases, the computational cost (in FLOPs) of the model decreases correspondingly. This is because the low-frequency branch operates at a lower resolution, resulting in reduced computational complexity. Because only the ratio α of the channels was changed, the Params for the networks corresponding to different α are the same. In terms of model performance, however, all evaluated metrics (including mAP@50:95, Precision@50/75, Recall@50/75, and F1-score@50/75) reach their optimal values when α=0.5. This outcome suggests that a balanced channel allocation between the high-frequency and low-frequency branches achieves the best trade-off between detection accuracy and computational efficiency. These findings are consistent with the theoretical insight that high-frequency and low-frequency information are complementary in visual representation: excessively favoring either frequency band weakens this complementary effect, thereby impairing the model’s capacity to capture and discriminate complex features, such as those corresponding to free fluid. Consequently, in subsequent experiments, we adopt the balanced configuration of α=0.5 as the default channel allocation scheme for the dual-branch architecture.

#### 4.2.2. Quantitative Performance Evaluation

To verify the effectiveness of the proposed modules, we conducted ablation experiments. The ablation experiments are based on YOLOx-tiny as the baseline model. First, the backbone and neck of YOLOx were individually replaced with the proposed components, which are labeled as DSF backbone and GFF neck, respectively. The complete integration of both modules is denoted as (DSF+GFF). Additionally, to validate the role of the dual-stream fusion mechanism in the DSF backbone, a control experiment was conducted in which the dual-branch structure was retained but inter-branch fusion was omitted. This variant is denoted as (No-fusion). The results of these ablation experiments are summarized in Table 3.

The experimental results demonstrate that compared with the baseline, employing the DSF backbone not only improves detection accuracy but also reduces FLOPs, confirming both the efficiency and performance benefits of the proposed dual-branch design. However, the use of a dual-stream structure leads to an increase in parameters. Using the GFF neck yields limited performance gains but effectively reduces computational complexity. The No-fusion variant further reduces computational cost relative to the full DSF backbone but exhibits a significant decline in performance, underscoring the critical role of inter-branch feature fusion in facilitating information complementarity and enhancing feature representation. In summary, the proposed DSF backbone and GFF neck each contribute meaningfully to improving model performance and reducing computational cost, with the strongest results achieved when both are used in combination. In summary, the DSF + GFF configuration achieves the highest performance across all evaluation metrics while maintaining the lowest computational cost in terms of FLOPs. Although its Params are not the lowest due to the dual-stream design, they remain lower than those of the baseline. For resource-constrained point-of-care devices, such a slight increase in storage overhead is acceptable, as computational efficiency is of greater concern in these scenarios.

#### 4.2.3. Qualitative Analysis of Intermediate Features

To further analyze the internal working mechanisms of the proposed modules, we conducted a visualization study on the intermediate features of YOLOx-tiny-Lite.

**Figure 8:**
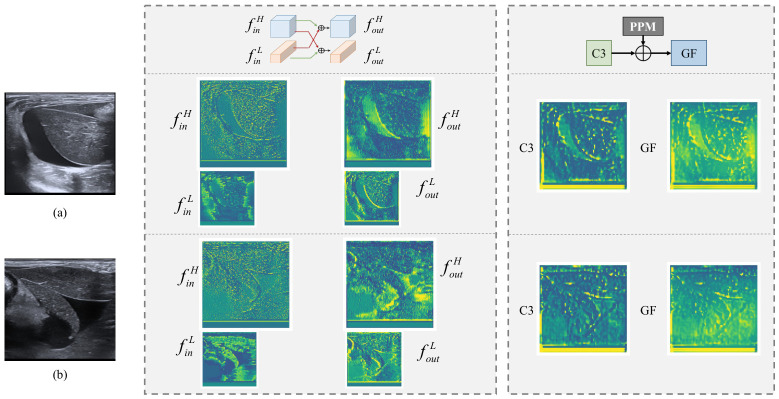
Visualization of feature maps from key modules in the proposed network. (**a**,**b**) illustrate two sample cases.

First, a cross-branch fusion mechanism based on the Octave Convolution is incorporated into the DSF backbone to integrate information from high- and low-frequency feature branches. To verify the effectiveness of this mechanism, we took the first Octave Convolution layer in the backbone as an example and visualized the feature maps of the two branches before and after fusion. As shown in Figure 8, before fusion, the high-frequency branch mainly captures detailed information such as edges and textures, while the low-frequency branch focuses on global structure and contour. After fusion, the high-frequency branch incorporates more structural information from the low-frequency branch, and the low-frequency branch shows enhanced high-frequency details. This indicates that the cross-branch fusion mechanism effectively enables the transfer and complementarity of different frequency information between the two branches, preventing information degradation or loss in either branch during feature extraction and thereby improving the completeness and discriminative power of feature representation.

Second, we visualized the generation process of the global fusion (GF) feature in the GFF neck. The GF feature is obtained by extracting multi-scale contextual information from high-level features via the Pyramid Pooling Module (PPM) and fusing it with the shallow feature C3. By comparing the pre-fusion C3 feature with the post-fusion GF feature, it is evident that the PPM-enhanced features exhibit significantly stronger response intensities in several local regions. These regions align closely in spatial distribution with the hypoechoic areas in the original ultrasound images—typical indicators of free fluid. This result demonstrates that the global contextual information extracted by the PPM module effectively highlights regions suspected of containing free fluid, enhances the model’s ability to model long-range semantic dependencies, and consequently improves detection robustness for targets such as free fluid, which exhibit variable morphology.

## 5. Discussion

This paper proposes a lightweight network for detecting free fluid in FAST examinations to screen for internal hemorrhage. Built upon YOLOx, the model introduces a dual-stream backbone that splits features into high-frequency and low-frequency branches according to ultrasound characteristics. The low-frequency branch captures the overall echo-intensity distribution and macro-shape of the fluid region with reduced computational cost, while the high-frequency branch provides details such as boundary sharpness and internal texture. To preserve information during feature extraction, a feature cross-fusion module is introduced after each stage to integrate both branches. For the neck module, a global fusion feedback mechanism aggregates multi-scale features into a unified global representation and feeds it back bottom-up, enhancing feature representation while reducing redundancy along the propagation path.

Experimental results demonstrate that the proposed method outperforms mainstream object detection methods in terms of detection accuracy, including precision, recall, and F1-score, while achieving the lowest computational complexity. YOLOx-tiny-Lite, constructed by incorporating the proposed lightweight strategy into the YOLOx-tiny baseline, only requires 3.543G FLOPs and 4.592M parameters, yet achieves the optimal mAP@50:95 of 71.02%. At an IoU threshold of 0.5, its precision, recall, and F1-score all exceed 98%; at IoU = 0.75, these metrics remain above 83%, indicating that the model can accurately detect free fluid and locate its position. YOLOx-nano-Lite, improved from the YOLOx-nano baseline with the proposed lightweight method, only consumes 0.554 G FLOPs and 0.82 M parameters, with its mAP@50:95 also higher than 70%. Further ablation studies show that the proposed DSF backbone reduces computational cost while improving detection accuracy, in which the cross-feature fusion module plays a key role in preventing information loss during dual-branch feature extraction. The GFF neck enhances the perception of free fluid regions through global feature fusion, further reducing computation while ensuring efficient multi-scale information fusion.

Although the above experiments have verified the effectiveness of the proposed lightweight method, the model deployment on edge hardware has not been implemented in this work. In future research, we will focus on operator optimization and software–hardware co-design, transplant the algorithm to portable ultrasound edge computing platforms such as FPGA/ARM, and carry out real-time inference tests and verification in clinical scenarios so as to further improve the engineering practical value of the proposed method.

The proposed model is primarily developed for intelligent FAST examination. Given the challenges in acquiring a sufficient number of ultrasound images of human internal hemorrhage and to more accurately capture the morphology of free fluid under active hemorrhage conditions, the model is trained using rabbit-derived ultrasound images from liver tissues with active hemorrhage. Rabbits exhibit high similarity to humans in terms of acoustic characteristics of free fluid in ultrasound imaging; training on this dataset, therefore, enables the model to more effectively extract sonographic features associated with active hemorrhage. Nevertheless, certain differences in organ morphology persist between rabbits and humans. While the dataset can be used to algorithmically validate the effectiveness of the proposed model, future research should focus on exploring effective strategies for model transfer and adaptation so as to facilitate its application in the actual diagnosis and treatment of human patients.

## 6. Conclusions

This paper proposes a lightweight network for free fluid detection in FAST examinations to screen for internal hemorrhage. The lightweight model significantly reduces computational costs while maintaining detection accuracy. Moreover, unlike prior studies, we used a clinically relevant dataset of ultrasound images from rabbits with active liver hemorrhage instead of static effusion data. The proposed method provides a feasible solution for free fluid detection on resource-constrained devices and advances the development of intelligent point-of-care assessment for internal hemorrhage.

## Figures and Tables

**Table 1:** Performance comparison with different methods. Upward arrows denote that higher values are preferable, and vice versa. Bold text indicates the use of the proposed lightweight method.

Method	Precision@50 ↑	Recall@50 ↑	F1-Score@50 ↑	Precision@75 ↑	Recall@75 ↑	F1-Score@75 ↑	mAP@50:95 ↑	FLOPs (G) ↓	Params (M) ↓
SSD	93.32	97.04	95.14	67.82	79.02	72.28	59.46	34.36	34.305
FCOS	95.15	99.20	97.15	78.20	86.60	82.20	64.90	38.83	19.094
Faster R-CNN	96.28	98.04	97.15	79.40	83.88	81.58	66.28	45.32	61.348
YOLOv3	96.02	98.58	97.26	71.34	80.06	75.45	64.74	50.00	61.949
YOLO26-n	97.61	98.77	98.19	82.09	85.91	83.96	69.87	3.950	2.365
YOLOx-s	97.58	98.76	98.16	82.24	86.34	84.22	69.98	8.524	8.938
YOLOx-tiny	96.92	98.58	97.76	81.04	85.50	83.20	69.32	4.845	5.033
YOLOx-nano	96.72	98.04	97.38	81.04	85.46	83.19	69.16	0.790	0.897
**YOLOx-tiny-Lite**	**97.96**	**99.12**	**98.36**	**82.94**	**87.08**	**85.06**	**70.82**	**3.543**	**4.592**
**YOLOx-nano-Lite**	**96.96**	**98.04**	**97.50**	**82.52**	**87.04**	**84.83**	**70.09**	**0.554**	**0.820**

**Table 2:** Performance comparison with different channel allocation ratio α in DSF-backbone. Upward arrows denote that higher values are preferable, and vice versa. Bold text indicates the optimal results.

α	Precision@50 ↑	Recall@50 ↑	F1-Score@50 ↑	Precision@75 ↑	Recall@75 ↑	F1-Score@75 ↑	mAP@50:95 ↑	FLOPs (G) ↓
0.2	96.92	98.60	97.74	80.74	85.66	83.04	70.20	4.692
0.4	97.14	98.76	97.94	81.64	86.38	83.94	70.56	3.951
0.5	**97.96**	**99.12**	**98.36**	**82.94**	**87.08**	**85.06**	**70.82**	3.543
0.6	97.10	98.22	97.66	81.26	85.84	83.46	70.34	3.400
0.8	96.94	98.22	97.58	80.62	85.20	82.90	68.44	**3.070**

**Table 3:** Quantitative results of ablation studies. Upward arrows denote that higher values are preferable, and vice versa. Bold text indicates the optimal results.

	Precision@50 ↑	Recall@50 ↑	F1-Score@50 ↑	Precision@75 ↑	Recall@75 ↑	F1-Score@75 ↑	mAP@50:95 ↑	FLOPs (G) ↓	Params (M) ↓
Baseline	96.92	98.58	97.76	81.04	85.50	83.20	69.32	4.845	5.033
No-fusion	97.06	98.58	97.80	81.12	85.06	83.06	68.94	3.582	**3.852**
DSF backbone	97.86	98.92	98.38	82.72	86.46	84.00	70.06	4.329	5.618
GFF neck	96.16	97.52	96.84	81.18	85.24	83.16	70.08	4.060	4.007
DSF + GFF	**97.96**	**99.12**	**98.36**	**82.94**	**87.08**	**85.06**	**70.82**	**3.543**	4.592

## Data Availability

The data presented in this study are available on request from the corresponding author due to privacy.
